# *In vivo* Environment Swiftly Restricts Human Pancreatic Progenitors Toward Mono-Hormonal Identity via a HNF1A/HNF4A Mechanism

**DOI:** 10.3389/fcell.2020.00109

**Published:** 2020-02-25

**Authors:** Thomas Aga Legøy, Andreas F. Mathisen, Zaidon Salim, Heidrun Vethe, Yngvild Bjørlykke, Shadab Abadpour, Joao A. Paulo, Hanne Scholz, Helge Ræder, Luiza Ghila, Simona Chera

**Affiliations:** ^1^Department of Clinical Science, University of Bergen, Bergen, Norway; ^2^Hybrid Technology Hub-Centre of Excellence, Faculty of Medicine, University of Oslo, Oslo, Norway; ^3^Department of Transplant Medicine, Institute for Surgical Research, Oslo University Hospital, Oslo, Norway; ^4^Department of Cell Biology, Harvard Medical School, Boston, MA, United States; ^5^Department of Pediatrics, Haukeland University Hospital, Bergen, Norway

**Keywords:** cell identity, cell fate, differentiation, endocrine progenitors, signaling, pathway analyses

## Abstract

Generating insulin-producing β-cells from human induced pluripotent stem cells is a promising cell replacement therapy for improving or curing insulin-dependent diabetes. The transplantation of end-stages differentiating cells into living hosts was demonstrated to improve β-cell maturation. Nevertheless, the cellular and molecular mechanisms outlining the transplanted cells’ response to the *in vivo* environment are still to be properly characterized. Here we use global proteomics and large-scale imaging techniques to demultiplex and filter the cellular processes and molecular signatures modulated by the immediate *in vivo* effect. We show that *in vivo* exposure swiftly confines *in vitro* generated human pancreatic progenitors to single hormone expression. The global proteome landscape of the transplanted cells was closer to native human islets, especially in regard to energy metabolism and redox balance. Moreover, our study indicates a possible link between these processes and certain epigenetic regulators involved in cell identity. Pathway analysis predicted HNF1A and HNF4A as key regulators controlling the *in vivo* islet-promoting response, with experimental evidence suggesting their involvement in confining islet cell fate following xeno-transplantation.

## Introduction

Loss of insulin-producing pancreatic β-cells ultimately characterize most diabetic conditions, prompting extensive efforts aimed at replenishing these cells from either endogenous (Chera and Herrera, 2016; Zhou and Melton, 2018) or exogenous sources (Balboa and Otonkoski, 2015). As part of the latter strategies, the employment of human induced pluripotent stem cells (hiPSC) as a source for insulin-producing cells has gained a lot of momentum in the recent years (Loo et al., 2018; Odorico et al., 2018). These methods are based on a stepwise hiPSC differentiation strategy mirroring the stages characterizing β-cells fate acquisition during development (Pagliuca and Melton, 2013; Melton, 2016; Petersen et al., 2018).

Despite generating insulin-producing cells with varying degrees of functional maturity, most current protocols present heterogeneous outputs, also producing cells secreting other pancreatic islet hormones (Petersen et al., 2017; Vethe et al., 2017). Moreover, some of the differentiating cells presents a hybrid phenotype, expressing more than one hormone (Bruin et al., 2014; Petersen et al., 2017; Vethe et al., 2017). This is in stark contrast to the *bona fide* human islet cells, which in homeostatic conditions are restricted to secrete a single pancreatic hormone: glucagon (α-cells), insulin (β-cells), somatostatin (δ-cells), pancreatic polypeptide (PP/γ-cells) or ghrelin (ε-cells) (Herrera, 2000; Desgraz and Herrera, 2009). The ambiguous hormone selection presented by the cells differentiated *in vitro* represents an important problem (Kushner et al., 2014), as this is usually connected with functional immaturity. Consequently, many differentiation protocols were aimed at improving the monohormonal cell fractions. Recent studies (Nair et al., 2019; Velazco-Cruz et al., 2019) report novel embryonic stem cells (ESC) differentiation strategies leading to substantial improvements of β-cell maturation and functionality. Indeed, these ESC-derived β-cells presented an energy metabolism fingerprint and glucose stimulated insulin secretion similar to the one observed in human islets.

In addition, xeno-transplantation into living hosts, such as mice, has been shown to significantly increase the yield and functionality of the differentiating hPS-derived cells (Kroon et al., 2008; Rezania et al., 2012, 2014; Pagliuca et al., 2014). Indeed, after extensive periods of time (2–6 months), the xenotransplantation of circa two million *in vitro* differentiated cells was able to normalize the glycemia in diabetic mice (Pagliuca et al., 2014; Rezania et al., 2014; Agulnick et al., 2015; Vegas et al., 2016; Bochenek et al., 2018; Saber et al., 2018). Although these experiments highlighted the importance of the *in vivo* environment and its systemic factors in promoting islet cell fate, the signals governing this process are largely unknown. Moreover, the graft response to the *in vivo* environment was not yet properly characterized.

In this study we aimed to address this knowledge gap by demultiplexing and characterizing the initial response of the hiPSC-derived differentiating pancreatic progenitors to the *in vivo* environment, using global proteomics and large-scale imaging techniques. Here we show that the *in vivo* exposure rapidly routes a large fraction of human pancreatic progenitors toward single hormone expression. Moreover, the overall proteome landscape of the transplanted cells was closer to a native islet-like regulation pattern and especially the energy metabolism and redox signature. Our study suggests a potential link between these, and the improvement of hormone selection through regulation of epigenetic factors involved in maintaining and propagating the patterns of hormone expression. Last, we identified by pathway analysis two upstream regulators, HNF1A and HNF4A predicted to be responsible for the *in vivo* islet promoting response of the transplanted cells and experimentally confirmed their role in confining human pancreatic progenitors to single hormone expression.

## Materials and Methods

### Cell Sources and Ethics Statements

The Norwegian Regional Committee of Medical and Health Research Ethics approved the reported experimental protocols used for hiPSCs (REK 2010/2295) and for human islets (REK 2011/426). All methods were carried out in accordance with the Helsinki Declaration. Informed consent was obtained from the healthy and MODY1/3 patient donors (skin fibroblasts) or from the relatives (organ donations). The human induced pluripotent stem cells (hiPSCs) used in this paper were generated using episomal reprograming with vectors from Addgene #27077 (OCT3/4), #27080 (L-MYC, LIN28) and #27078 (SOX2, KLF4) as previously described by us (Vethe et al., 2017; Bjørlykke et al., 2019). Proteomic analyses of *HNF1*α^Δ/+^ was performed on cell lines generated by Sendai reprograming carried out by Tekara Bio Inc., using a CytoTune-iPS 2.0 Sendai reprogramming kit (#A16517, Life Technologies) as described in Bjørlykke et al. (2019). All hiPSC lines were negative for mycoplasma, by using MycoAlert Mycoplasm Detection Kit (Lonza, LT07-418). Seven distinct iPSC lines were used in the study. The pluripotency of the iPSC lines was previously tested (Vethe et al., 2017; Bjørlykke et al., 2019), while their differentiation potential was previously assessed in Vethe et al. (2017), Legoy et al. (2019), Vethe et al. (2019a). Human islets were obtained as previously described (Friberg et al., 2008) from seven deceased donors (males and females).

### Cell Preparation

The hiPSC lines were enriched for SSEA4^+^ cells using magnetic beads (#130097855 MACS Miltenyi Biotec) before *in vitro* differentiation. Both normal and mutated hiPSCs were differentiated according to a seven-stage protocol (Rezania et al., 2014). The planar differentiation efficiencies estimated as insulin^+^ NKX6.1^+^ co-expressing cells were similar with the previously reported values (Supplementary Figure S1A). Also, this percentage was similar between WT and HNF1A^Δ/+^ in two independent differentiation rounds (Supplementary Figure S2L). The differentiation efficiencies for HNF4A^Δ/+^ clones was previously assessed in Vethe et al. (2017) and consequently assumed similar in this work. For this study we used 2D differentiation on Matrigel-coated plates until Stage 5 (S5; pancreatic endocrine precursors), or up to Stage 7 (S7; maturing beta-cells), S5 cells were encapsulated in alginate before continued differentiation toward S7, and S5 and S7 cells encapsulated in alginate prior to *in vivo* transplantation. Alginate encapsulation was performed as we have described earlier (Vethe et al., 2019b). Cell number and viability was measured as previously described (Vethe et al., 2019b). Cell viability mean was 86.53 ± 7.03%. The fraction of dying cells at different time points following transplantation in normoglycemic animals was similar to the previously reported (Legoy et al., 2019) and was estimated at ∼7.5% between 1- and 4-weeks postTX.

### Flow Cytometry Analysis

Differentiating cells from stage 5 (pancreatic endocrine precursors) or stage 7 (maturing beta cells), were collected using TrypleTM Select Enzyme (1X). Cells were first checked for viability using NucleoCounter^®^ NC-200^TM^ (Dąbrowskiego, Poznań, Poland), and only more than 80% viable cells were used in the assay. Cells were washed and incubated in a 96-well plate (v-bottom, 2 × 105 cells per well) with Fixation/Permeabilization solution 20 min. After washing with Perm/Wash Buffer, cells were stained for 45 min in the dark at RT with FITC-conjugated anti-insulin and AF647-conjugated anti-NKX6.1. Markers were set according to the isotype control FITC or AF647-conjugated mouse IgG1. After washing in PBS, the cells were examined on a flow cytometer (BD LSRFortessa, Becton-Dickinson Biosciences, San Jose, CA, United States). Gating was performed according to the isotype control. FACS analysis was done using FlowJo (Flow cytometry analysis software, Ashland, OR, United States).

### Transplantation

For transplantation we used the following transgenic mouse line NOD.*Cg-Prkdc^*scid*^Il2rg^*tm1Wjl*^* Tg (Ins2-HBEGF) 6832Ugfm/Sz (Yang et al., 2015) referred to as NSG RIP-DTR. Three mice (8–12 weeks old) were used for each group. The mice for the diabetic group received diphtheria toxin injections intraperitoneal (ip) as previously described (Thorel et al., 2010; Cigliola et al., 2018). Glycemia were measured two times per week with a Contour XT glucometer (Bayer). The mice received transplantation of alginate beads intraperitoneally when anesthetized with inhalable sevoflurane administered via Datex-Ohmeda Sevotec 5. Each mouse was transplanted with approximately five million cells. Transplanted mice received 0.5 mg/ml paracetamol in the drinking water for 5 days post-transplant. At the end of *in vivo* incubation of the transplanted cells, mice were euthanized by cervical dislocation and the alginate beads were collected from the intraperitoneal cavity by lavage. Beads were rapidly washed in saline solution for removing traces of blood and host tissue and either immediately fixed in 4% PFA or transferred in lysis buffer for proteomics. All animal procedures were performed in accordance with the EU Directive 2010/63/EU for animal experiments. The breeding strategy and experimental protocols were approved by the Norwegian Animal Research Authority (FOTS IDs 8329 and 8423). The mice were housed in individually ventilated cages (IVC) enriched with wooden bedding, nesting material, in a temperature-controlled environment at 22°C under a 12-h light-dark cycle. The mice were given *ad libitum* access to water and standard diet RM1A (SDS).

### Immunofluorescence

Preparation and immunofluorescence of alginate beads were performed as previously described (Vethe et al., 2019b). The pancreas from the mice were fixed for 2 h in 4% PFA, before dehydration using a sucrose gradient of 10, 20, and 30% and embedded in Tissue Tek OCT compound (Sakura JP). Sections of 10 μm were obtained with a cryotome (Leica CM 1950, Leica, DE) and added on SuperFrost Plus slides (Thermo Scientific). The immunofluorescence staining was performed following indications provided by the supplier. The following primary antibodies were used: mouse anti-insulin IgG1 (1/500, I2018, Sigma-Aldrich), guinea-pig anti-porcine insulin (1/400, A056401-2, Dako), mouse anti-porcine glucagon (1/1000, G2654, Sigma-Aldrich). The following secondary antibodies were used at dilution 1/500: goat anti-mouse IgG1 A488, goat anti-guinea-pig A488, goat anti-guinea-pig A546, goat anti-mouse IgG1 A546. DAPI (1/1000, D1306, Molecular Probes) was used to stain the nuclei. The samples were mounted in Prolong Diamond Antifade Mountant Media (P36970, Life technologies). Image acquisition and analysis was performed on Andor Dragonfly confocal microscope and Imaris 9.1.2 (Bitplane AG) as we have previously described (Vethe et al., 2019b). We also performed manual counting on images acquired using a 40x immersion objective on Leica TCS SP5 confocal (Leica Microsystems CMS GmbH).

### Global Proteomics Analysis

The proteomics analysis for the *in vitro* samples and associated human islets were performed as we have earlier described (Vethe et al., 2019b) dataset identifier PXD012704, with the exception of the *HNF1*α^Δ/+^ samples which were performed as in Vethe et al. (2017). Protein samples from the *in vivo* transplanted cells were processed for Tandem Mass Tag (TMT) 11-plex as for the *in vitro* samples, apart from the use of halved volume of TMT reagents. A total of 12 fractions were collected from the pooled TMT-labeled peptide samples using the Pierce High pH Reversed-Phase Peptide Fractionation Kit (cat. # 84868) and 7.5, 10, 12.5, 15, 17.5, 20, 22.5, 25, 27.5, 30, 35, and 60% acetonitrile. Every sixth of these fractions were combined to yield six fractions in total. Subsequently we acidified the samples using 1% formic acid before vacuum centrifuged to near dryness. Each fraction was desalted using StageTip, dried via vacuum centrifugation, and reconstituted for LC-MS/MS processing. For LC-MS3 analysis ∼5 μg from each fraction was dissolved in 15% aqueous formic acid/5% acetonitrile prior to loading on an Orbitrap Fusion Lumos mass spectrometer (Thermo Fisher Scientific, San Jose, CA, United States) coupled to a Proxeon EASY-nLC 1200 liquid chromatography (LC) pump (Thermo Fisher Scientific). By using a 100-μm inner diameter microcapillary column packed with ∼35 cm of Accucore resin (2.6 μm, 150 Å, ThermoFisher Scientific) the peptides were fractionated. We loaded ∼1 μg onto the column for each analysis. Subsequent separation and acquisition were performed as previously described (Vethe et al., 2019b). Samples were analyzed in duplicate, one with advanced peak determination (ADP) activated and a second run with this option off. Both analyses used the real-time search (RTS) algorithm (Erickson et al., 2019). Data was searched against the UniProt human database (downloaded: October, 2016). The dataset was uploaded to ProteomeXchange via the PRIDE^1^ partner repository with the dataset identifier PXD015955. The hyperglycemia samples from this dataset were not analyzed in this work. Two human islets samples were excluded from analysis due to islet quality problems.

### Proteomic Data Analysis

We analyzed the mass spectrometry data as earlier described (Vethe et al., 2019b). Protein quantify values were analyzed using Microsoft Excel and GraphPad Prism (version 8). Hierarchical clustering was performed using with GeneSpring 14.9.1 GX software (Agilent), with clustering on both entities and conditions by using Squared Euclidian distance metric and Ward’s linkage rule. The pathway analyses were generated by QIAGEN’s Ingenuity Pathway Analysis program (IPA^®^, QIAGEN, Redwood City, United States)^2^ (Kramer et al., 2014) as previously described (Vethe et al., 2019b), here using 35 molecules/network; 25 networks/analysis for generating the interaction networks.

### Statistical Analysis

For the immunofluorescence counting we used Mann-Whitney non-parametric test to compare the number of hormonal cells between the different groups using GraphPad Prism v8.1.2. Also, unpaired two-tailed Student’s *t*-tests were used for analyzing the proteomics data. A *p*-value of <0.05 was considered significant.

## Results

### Short-Term Exposure of Encapsulated Pancreas Progenitor Cells to *in vivo* Environment Promotes an Islet-Like Signature

To investigate the short-term effect of the *in vivo* environment on islet cell differentiation, we xenotransplanted encapsulated hiPSC-derived pancreas progenitors (from here on S5-cells) into the intraperitoneal cavity of humanized NSG mice (Supplementary Figure S1B). Based on its consistent reproducibility and popularity, we employed an established protocol (Rezania et al., 2014) for the initial directed differentiation (*in vitro*). The resulting S5-cells (five million cells) were encapsulated in alginate and then immediately transplanted into normoglycemic mice for 2 weeks (*in vivo*), a period of time equivalent to the one required for generating maturing islet cells *in vitro* (2 weeks).

**FIGURE 1:**
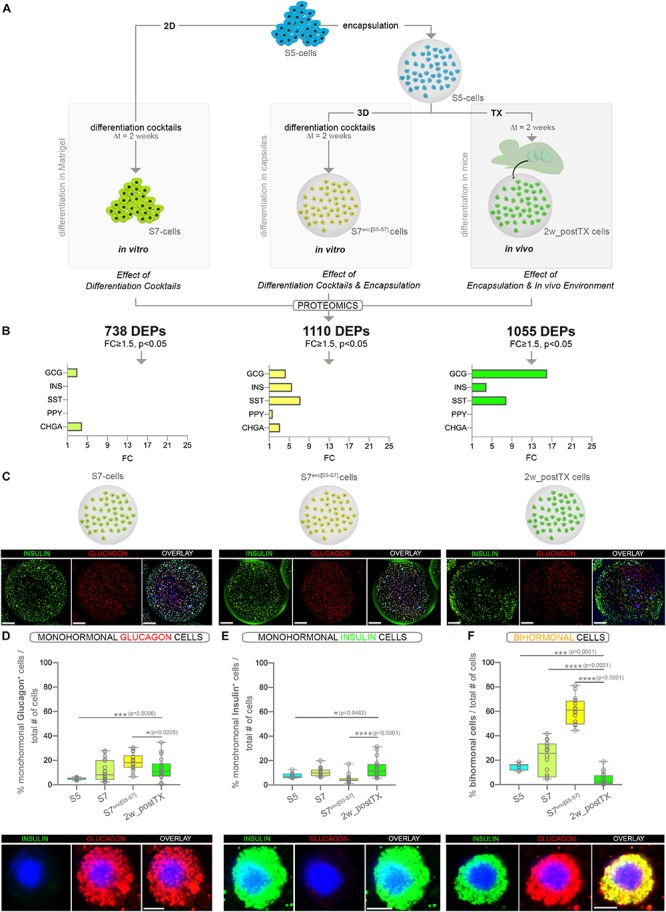
Assessment of the pancreatic islet hormones expression by global proteomics and large-scale imaging following three distinct differentiation strategies **(A)** Experimental design depicting the three differentiation strategies considered. Numbers in bold represent differentially expressed proteins (DEPs) between the final differentiation stage (S7, S7^enc[S5–S7]^, 2w_postTX) and pancreatic progenitors (S5), following islet-standard normalization (data represent two distinct TMT-11 plexes). **(B)** Graphs displaying the statistically significant (FC ≥ 1.5, *p* < 0.05) regulated islet hormones (colored bars) identified in the DEPs set characterizing each differentiation strategy (*in vitro* Matrigel differentiation – chartreuse, *in vitro* encapsulation – yellow and *in vivo* transplantation of encapsulated cells – light green). **(C)** 3D reconstructions of dragonfly imaged whole alginate capsules containing cells immunofluorescently labeled for insulin (green), glucagon (red) and DAPI (blue) in the four conditions analyzed (scale bar 200 μm, gamma correction 0.4). **(D)** Proportion of monohormonal glucagon cells in the four distinct populations analyzed (*n* = 9,18,18,18) and the high magnification of a representative encapsulated glucagon^+^ cell. **(E)** Proportion of monohormonal insulin in the four distinct populations analyzed (*n* = 9,18,18,18) and the high magnification of a representative encapsulated insulin^+^ cell. **(F)** Proportion of bihormonal cells (insulin^+^glucagon^+^ co-expressing) and a high magnification of a representative encapsulated bihormonal cell. High magnification scale bars: 5 μm. Graphs data are shown as box plot mean to max values. *p < 0.05, **p < 0.01, ***p < 0.001, ****p < 0.0001 (Mann–Whitney test). Abbreviations: TX, transplant; 2D, Matrigel differentiation; 3D, alginate encapsulation; DEPs, differentially expressed proteins; Dt, time interval; FC, fold change; S5-cells, stage 5 cells (pancreatic progenitor stage); S7-cells, stage 7 cells (maturing β-cells); S7^enc[S5–S7]^, differentiated in capsules from stage 5 to stage 7; 2w_postTX, 2 weeks following transplantation (differentiation in mice).

As the mechanical forces elicited by the encapsulation of cells can in itself interact with the differentiation response to the differentiation cocktails (Vethe et al., 2019b), we used an array of experimental conditions aimed to ultimately demultiplex the *in vivo*-specific molecular mechanisms stimulating the islet cell fate acquisition program (Figure 1A). As such, pancreas progenitors (S5-cells) were differentiated toward islet cell fates (1) *in vitro* on Matrigel-coated plates (planar 2D culture condition; termed “S7-cells”), (2) *in vitro* in alginate capsules (termed “S7^enc[S5–S7]^″) and (3) *in vivo* following xenotransplantation (termed “2w_postTX”). In this setup, the comparative analysis of the molecular signatures exhibited by the resulted end-stage cellular entities grants the possibility of discriminating between the effects of the (i) differentiation cocktail, (ii) encapsulation, (iii) *in vivo* environment.

We first performed global proteomics on the S5 cells, their respective differentiation counterparts (S7, S7^enc[S5–S7]^, 2w_postTX cells) and human islets isolated from cadaveric donors. 2369 proteins were detected in all conditions across TMT-plexes (Supplementary Figure S1C). About two thirds (64.83%, 1536/2369) represented differentially expressed proteins (DEPs) between the S5-cells (pancreatic progenitors) and the directed differentiation goal, the bona fide human islet cells (FC ≥ 1.5, *p* < 0.05). The principal component analysis (PCA) revealed a clear separation of the samples according to the differentiation strategy (Principal Condition 1: 40.48%). The 2w_postTX cluster of samples exhibited the highest similarity with human islets (Supplementary Figure S1D, green). The hierarchical clustering confirmed these results, as the recovered xenotransplanted cells branched closer to human islets (Supplementary Figure S1E). Furthermore, a direct comparison of these sets revealed that 14.78% (227/1536) proteins were differentially expressed between transplanted cells and islets. These results indicate that a relevant number of proteins changed their pattern of expression toward an islet-like signature even after just a brief exposure to the *in vivo* environment.

### Short-Term Exposure to *in vivo* Environment Increases the Abundance of the Main Islet Hormones

Based on their islet-normalized profile, 738, 1110 and respectively, 1055 proteins exhibited a significant change of their regulatory dynamic (FC ≥ 1.5, *p* < 0.05) in response to the distinct differentiation conditions described above (Figure 1A, Supplementary Figure S1F, and Supplementary Table S1). Among the 1055 DEPs regulated following 2 weeks of transplantation, the main islet-specific hormones, insulin, glucagon and somatostatin were significantly upregulated, with glucagon displaying the highest increase (Figure 1B). Interestingly, glucagon expression was promoted by all differentiation conditions, while insulin and somatostatin were promoted only by the 3D settings (*in vitro* and *in vivo* encapsulated conditions). In contrast, the pancreatic polypeptide, a hormone produced by the pancreatic PP/γ-cells, was not promoted in the transplanted samples, being significantly regulated solely in the *in vitro* differentiating capsules (S7^enc[S5–S7]^). Last, the pan-endocrine marker Chromogranin A (CHGA), which exhibited increased abundance in the *in vitro* setups, was not significantly regulated *in vivo* (Figure 1B).

These data suggest that even short exposure of S5 cells to the *in vivo* environment is enough to increase the expression of the three main islet hormones, including insulin. Nevertheless, it is not sufficient for induction of pan-endocrine markers such as CHGA. This indicates that the brief exposure to the *in vivo* environment promotes hormone production in the differentiating cells, but is probably insufficient for recruiting new progenitors toward the endocrine program.

### Short-Term Exposure to *in vivo* Environment Does Not Increase the Hormone Positive Fraction but Improves Their Monohormonal Identity

To discriminate if the brief *in vivo* exposure increases the fraction of hormone positive cells as opposed to improving the hormone production in the transplanted encapsulated cells, we performed large scale imaging microscopy of the three differentiation conditions. To allow the use of similar counting algorithms, a similar number of the cells differentiated in the planar 2D setup (S7-cells) were fixed and encapsulated just before image acquisition (Figure 1C). Briefly, for each capsule a mosaic of nine fields of view (FoV) over 100 *z*-planes were acquired, reconstructed and automatically quantified by using Imaris 9.1.2 as previously described by us (Vethe et al., 2019b). The quantification revealed the rise of the monohormonal glucagon^+^ (Figure 1D) and insulin^+^ (Figure 1E) fraction in the encapsulated cells following brief exposure to the *in vivo* environment (compare 2w_postTX and S5 columns). Moreover, this was coupled with a significant decrease in the proportion of immature bihormonal cells (Figure 1F). Overall, these results corroborate that rather than increasing the number of hormone expressing cells, the *in vivo* transplantation enhances the quality of the differentiated encapsulated cells. This is probably achieved by refining the accuracy of cell fate/hormone status selection and improving hormone production.

### The Demultiplexed *in vivo* Effect Promotes Oxidative Phosphorylation and Neuritogenesis

Further, to globally characterize the response to the *in vivo* environment besides islet hormone regulation, we first discriminated the *in vivo* effect signature and subsequently performed pathway analysis.

**FIGURE 2:**
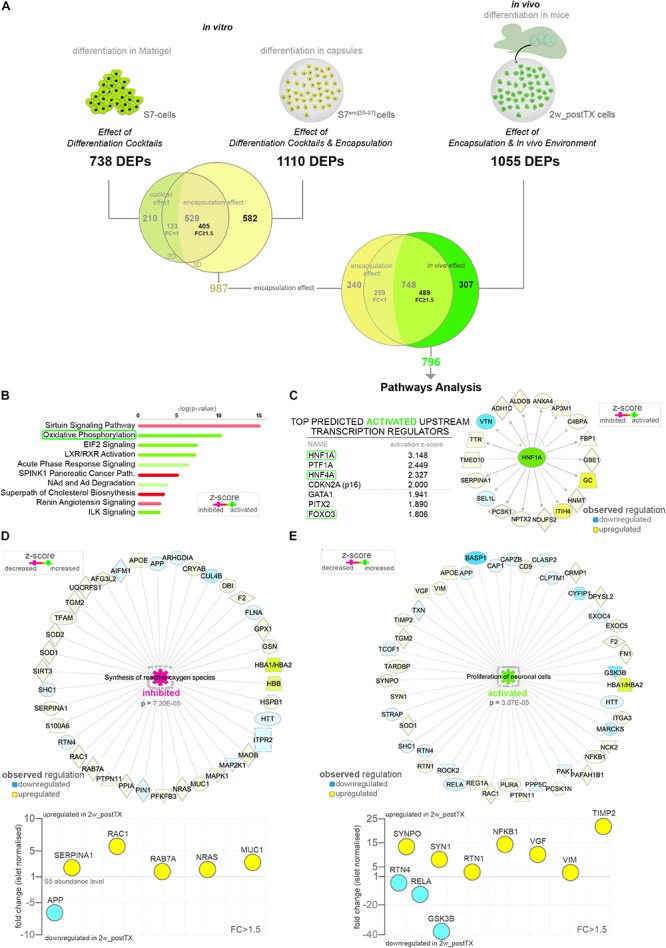
Pathway analysis of the proteome landscape following *in vivo* exposure. **(A)** Analysis workflow depicting the strategy used for demultiplexing the *in vivo* effect **(B)** IPA-generated top canonical pathways with predicted regulation (*z*-score ≥ 1.1) characterizing the *in vivo* response. **(C)** Top predicted activated upstream transcription regulators and the HNF1A target molecules observed regulated in the *in vivo* effect DEPs dataset. **(D,E)** IPA-generated networks and graph representations of selected dataset DEPs characterizing the corresponding top disease and function processes. Abbreviations: TX, transplant; DEPs, differentially expressed proteins; FC, fold change; S5-cells, stage 5 cells (pancreatic progenitor stage); S7-cells, stage 7 cells (maturing β-cells); S7^enc[S5–S7]^, differentiated in capsules from stage 5 to stage 7; 2w_postTX, 2 weeks following transplantation (differentiation in mice), Path, pathway. For abbreviations see Supplementary Table S3.

As the S5-cells are encapsulated before transplantation to protect against the immune attack as well as to allow their retrieval, the above results reflect the combinatorial impact of both alginate encapsulation and *in vivo* environment. To focus exclusively on the latter (*in vivo* effect), the readout of the three differentiation setups was used to accurately exclude the encapsulation effect. First, to comprehensively define the protein set significantly regulated by encapsulation, we compared the two *in vitro* conditions, i.e. standard differentiation (differentiation cocktail effect) and cells differentiating in capsules (differentiation cocktail and encapsulation effect) (Figure 2A). The Venn comparison revealed 582 DEPs being modulated exclusively as a result of the encapsulation effect. Moreover, although 528 DEPs were found regulated by both conditions, about three quarters (76.70%, 405/528 DEPs) displayed distinct regulatory patterns according to the differentiation setup (FC ≥ 1.5 of difference). This indicates that despite being mainly targets of the differentiation cocktail, their regulation is also significantly influenced by encapsulation and thus likewise a consequence of this effect. Subsequently, the overall encapsulation effect signature was compared against the *in vivo* differentiation setup. The Venn diagram filtered 307 DEPs regulated solely by the *in vivo* effect, while 489 shared a common, however, differential, regulation of the encapsulation and *in vivo* effects (Figure 2A and Supplementary Table S2).

To identify the protein networks, signaling pathways and upstream regulators characterizing the overall *in vivo* environment signature, we performed pathway analysis on the resulted *in vivo* effect protein set (Supplementary Table S2). The Ingenuity Pathway Analysis (IPA) software revealed a strong energy metabolism (Supplementary Figure S2A), with pathways such as oxidative phosphorylation (OXPHOS) being confidently predicted as activated following transplantation (Figure 2B). This is suggestive of the impact of transplantation on the redox balance of the differentiating cells. Furthermore, the analysis identified the Hepatocyte nuclear factor 1-alpha (HNF1A) transcription factor as the leading activated upstream regulator responsible for the observed regulatory landscape (Supplementary Figure S2B). Of note, in the top activated transcriptional regulators the program inferred the involvement of transcription factors involved in the pancreatic islet development (Hepatocyte nuclear Factor 4A – HNF4A and Pancreas transcription factor 1A – PTF1A), as well as cellular senescence and growth (Cyclin-dependent kinase inhibitor 2A – CDKN2A and Forkhead box protein O3 - FOXO3), suggesting the role of the *in vivo* niche on islet cell fate decision, consistent with the above observations (Figure 2C and Supplementary Figure S2C).

Interestingly, the disease and function analysis pinpointed with high confidence the decrease of both synthesis and production of active oxygen species (Figure 2D and Supplementary Figure S2D), which combined with the observed increase of OXPHOS and low antioxidant activity (Figure 2B), advocate a scenario consistent with an improvement of the energy metabolism of the differentiating cells following transplantation and not a tilt of the redox balance toward oxidative stress.

Aside from the energy metabolism signature, the analysis predicted a decrease in the cholesterol metabolism and an activation of vitamin metabolism in the transplanted cells (Supplementary Figure S2D). Moreover, a robust activation of a neurogenesis/neuritogenesis signature was supported by a large fraction of the data set (Figure 2E and Supplementary Figure S2E), which combined with the short transplantation period argue in favor of the transplanted differentiating cells (graft) stimulating the innervation from the niche. This is similar to islet cells transplanted underneath the kidney capsule, known to promote the innervation of the graft in the first weeks following transplantation (Korsgren et al., 1993; Tang et al., 2014).

### The *in vivo* Environment Shifts the Energy Metabolism Signature of the Transplanted Cells Toward Native Islet Regulation

**FIGURE 3:**
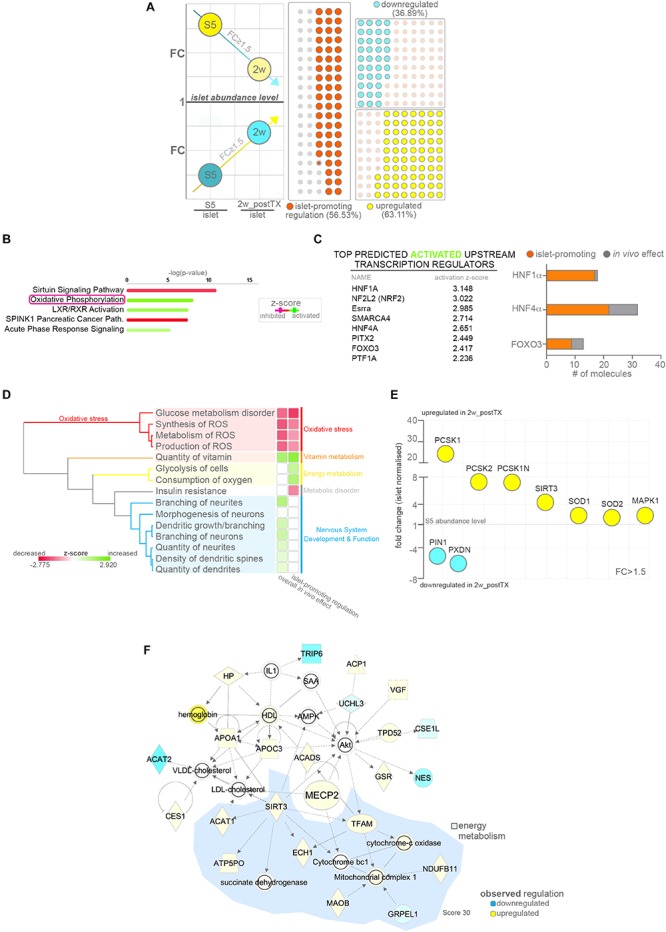
Pathway analysis of proteins following islet-promoting regulation patterns as result of the *in vivo* effect. **(A)** Scheme depicting the selection strategy and waffle charts reflecting the number of proteins showing a dynamic of regulations compatible with an islet-promoting pattern in response to the *in vivo* effect. The scheme circles reflect the regulation reported to the islet abundance levels. Arrows represent the generic mandatory direction of regulation for inclusion in the islet-promoting signature (blue – downregulation, yellow – upregulation). The waffle graph dots reflect the regulation dynamic following transplantation (similar to the scheme arrows) **(B)** IPA-generated top canonical pathways with predicted regulation (*z*-score ≥ 1.8) characterizing the protein subset exhibiting islet-promoting regulation. **(C)** Top predicted activated upstream transcription regulators and graph depicting the number of relevant target DEPs in the HNF1A, HNF4A and FOXO3 network following a regulation toward islet abundance levels. **(D)** IPA generated hierarchical clustering of the predicted disease and function processes for the conditions compared. **(E)** Selected signature-relevant DEPs exhibiting regulation toward islet abundance levels. **(F)** Selected top 5 organic network linking MECP2, lipid and energy metabolism. Abbreviations: DEPs, differentially expressed proteins; FC, fold change; S5, stage 5 cells (pancreatic progenitor stage); 2w_postTX, 2 weeks following transplantation (differentiation in mice), Path, pathway; ROS, reactive oxygen species). For abbreviations see Supplementary Table S3.

To assess if the observed proteome landscape changes induced by the *in vivo* effect lead to an improved islet-like signature, we focused on differentially expressed proteins that following *in vivo* exposure are regulated toward the abundance levels normally detected in native islets (Figure 3A). About half of the proteins encompassed by the *in vivo* effect (56.53% 450/796, Supplementary Table S2) presented an islet-promoting regulation, with ∼22.23% (177/796) reaching islet abundance values. Most (63.11% 284/450) were upregulated, i.e. characterized by low abundance in S5-cells, as compared to islets followed by an increase toward islet abundance levels following transplantation (Figure 3A, lower quadrant).

The pathway analysis of the protein subset exhibiting islet-promoting regulation revealed that the proteins defining the energy metabolism signature detected above are encompassed in this group (Figure 3B and Supplementary Figure S2F). This suggests that transplantation improves the energetic status of the cells toward the one presented by native islets. The evaluation of the observed regulatory landscape pointed once again to HNF1A as top activated upstream regulator (Supplementary Figure S2G), while RICTOR (Rapamycin-insensitive companion of mTOR) was predicted as top inhibited (Supplementary Figure S2H). In addition, the activation of transcription factors with function in islet development and growth, such as HNF4A or FOXO3, was reiterated in this context, suggesting that the protein signatures defined by these regulators contribute to the acquisition of an islet-like phenotype. Indeed, the vast majority of the proteins compatible with HNF1A (94.44%), HNF4A (68.75%) and FOXO3 (69.23%) regulation responded to the *in vivo* effect by following an islet-promoting regulation (Figure 3C and Supplementary Figure S2I).

Correspondingly, the comparative overall disease and function analysis inferred the signature of oxidative stress inhibition to be orchestrated by proteins displaying an islet-promoting regulation in response to the *in vivo* environment (Figure 3D, red cassette). Moreover, an active glycolysis and oxygen consumption was also predicted based on the proteins belonging to this subset, strengthening the assertion of an improved energetic and metabolic status of the cells following transplantation (Figure 3D, yellow cassette). In contrast, the neurogenesis/neuritogenesis signature identified in the overall *in vivo* effect was not detected in the islet-promoting subset (Figure 3D, blue cassette), indicating that the proteins characterizing this signature do not contribute directly to the acquisition of an islet-like cell fate, rather fulfill a maintenance role required for the graft adaptation and survival in the transplantation niche.

Of note, Neuroendocrine convertase 1 (PCSK1, +24.30×, *p* = 0.0002) and 2 (PCSK2, +7.31×, *p* = 0.0073) the key neuroendocrine convertases responsible for insulin, glucagon and somatostatin processing were amongst the significantly upregulated proteins displaying a sustained islet-promoting regulation toward values presented in native islets. Similarly, the levels of SOD1 (superoxide dismutase 1, +2.372× *p* = 0.0177) and SOD2 (superoxide dismutase 2, +2.068×, *p* = 0.0074), essential for destructing superoxide radicals and maintaining the cell redox balance, displayed improved abundance levels, closer to the ones detected in human islets. Finally, proteins involved in synthesis of reactive oxygen species, such as PIN1 (Peptidyl-prolyl *cis-trans* isomerase NIMA-interacting 1, −5.08×, *p* = 0.0007) and PXDN (Peroxidasin homolog, −6.05×, *p* = 0.0430) presented a steep decrease toward islet abundance levels following transplantation (Figure 3E).

Overall, these results suggest an improvement of the redox balance, glucose and energy metabolism in the transplanted cells in response to *in vivo* exposure. In contrast, other processes stimulated by the *in vivo* niche, such as neuritogenesis, do not follow a trend compatible to the islet cell fate acquisition, although they might, however, have an essential role in the graft survival.

### Improved Energy Metabolism Status Is Linked to Enhanced Expression of Epigenetic Modifiers Involved in Cell Fate Restriction

To investigate if the improved energetic signature could be related to the observed improvement of cell fate selection in the transplanted cells, we performed network analysis on the protein subset exhibiting islet-promoting regulation with a focus on modulated epigenetic modifiers. We identified in the dataset four DEPs with known epigenetic role (Supplementary Figure S2J). Further inquiry revealed the upregulation of MECP2 (Methyl-CpG-binding protein 2, 1.74×, *p* = 0.0458) in the *in vivo* exposed cells, a key enzyme involved in maintaining the repression of α-cell fate in β-cells (Dhawan et al., 2011). This enzyme acts by binding the methylated locus of the essential α-cell determinant gene, *ARX* (Aristaless-related homeobox), in β-cells, thus preventing its expression in the insulin producing cells. Previous studies demonstrated the importance of MECP2 for restricting hormone selection as its loss results in misexpression of key α-cell markers such as MAFB (V-maf musculoaponeurotic fibrosarcoma oncogene homolog) or glucagon in murine β-cells. Consequently, the detected improved expression of this enzyme is consistent with the observed decrease of the bihormonal fraction. Unfortunately, due to inherent sensitivity issues related to performing proteomics on encapsulated samples, we were unable to confirm ARX or MAFB decrease as a consequence of MECP2 activation in the transplanted cells. Of note, as MAFB retains its expression in mature human β-cells (Conrad et al., 2016; Cyphert et al., 2019), it is possible that its regulation is not affected in the human context. Interestingly, MECP2 was organically networked with regulated proteins involved in the lipid and energy metabolism (Figure 3F), suggesting a link between the improved energetic status of the transplanted cells and its upregulation.

### The *in vivo* Exposure Effect on Cell Fate Restriction Is Mediated by HNF1A and HNF4A Induction

Since the above analyses predicted a central role for HNF1A for the observed proteome landscape response to *in vivo* exposure, we focused on validating its role in islet cell fate decisions following transplantation. As to our knowledge there are no available pharmacological agents for modulating HNF1A activity, we employed hiPSC cells derived from Norwegian patients bearing a dominant heterozygous mutation (hot-spot mutation P291fsinsC) in the *HNF1A* (Bjorkhaug et al., 2003).

**FIGURE 4:**
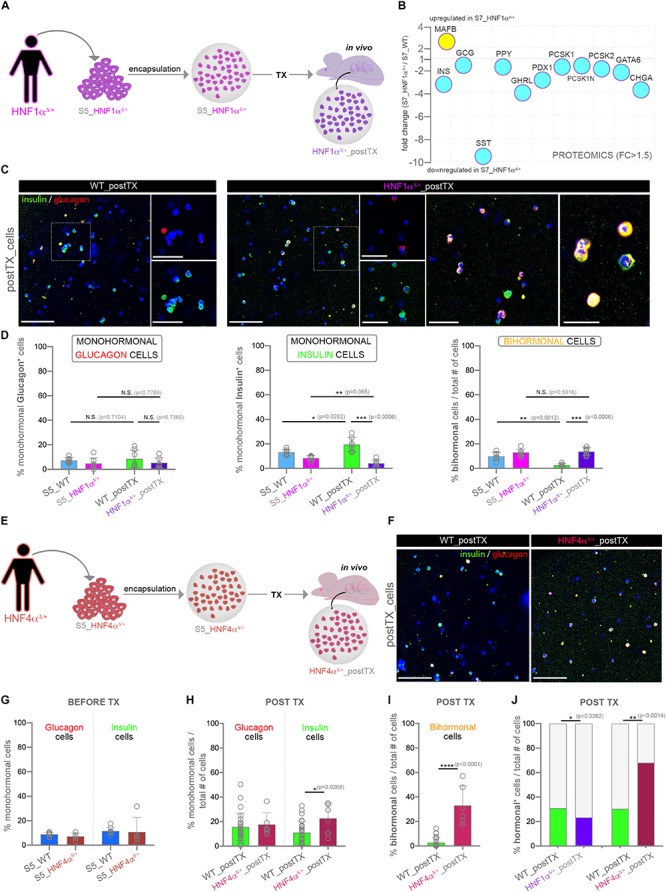
Assessment of hormone expression patterns in differentiating cells characterized by suboptimal HNF1A or HNF4A levels. **(A)** Experimental workflow. **(B)** Selected islet cell markers regulation in HNF1α^Δ/+^ (pool) following standard *in vitro* differentiation, identified by a pilot global proteomics experiment. **(C)** Confocal imaging of insulin (green), glucagon (red) and DAPI (blue) immunofluorescence staining of encapsulated *HNF1*α^Δ/+^ and WT cells following transplantation. **(D)** Manual counting of the proportion of monohormonal glucagon^+^ cells, monohormonal insulin^+^ cells and bihormonal glucagon^+^insulin^+^ in encapsulated *HNF1*α^Δ/+^ and WT samples before and after *in vivo* exposure (*n* = 7,8,8,7). **(E)** Experimental workflow. **(F)** Confocal imaging of insulin (green), glucagon (red) and DAPI (blue) immunofluorescence staining of encapsulated *HNF1*α^Δ/+^ and WT cells following transplantation. **(G,H)** The proportion of monohormonal glucagon + and monohormonal insulin + cells in *HNF4*α^Δ/+^ and WT samples **(G)** before (*n* = 9,5,9,5) and after **(H)** transplantation (*n* = 26,6,26,6) quantified by Imaris software. **(I)** The fraction of bihormonal glucagon^+^ insulin^+^ cells in HNF4α^Δ/+^ and WT samples after transplantation (*n* = 26,6). **(J)** The fraction of total hormonal^+^ cells in WT, HNF1α^Δ/+^ and HNF4α^Δ/ +^ samples after transplantation. Scale bars: 100 μm and 50 μm (high magnifications). Graphs data are shown as mean and SD. *p < 0.05, **p < 0.01, ***p < 0.001, ****p < 0.0001 (Mann–Whitney test). Abbreviations: DEPs, differentially expressed proteins; FC, fold change; WT, wild type; D, mutated allele; S5_WT, control stage 5 cells; S5_HNF1α, Stage 5 cells bearing the HNF1α^Δ/*WT*^ mutation; WT_postTX, control cells following transplantation, HNF1α^Δ/*WT*^ _postTX, HNF1α^Δ/*WT*^ cells after transplant; S5_HNF4α, Stage 5 cells bearing the HNF4α^Δ/*WT*^ mutation; HNF1α^Δ/*WT*^ _postTX, HNF1α^Δ/*WT*^ cells after transplant.

We first differentiated *HNF1*α^Δ/+^ hiPSC toward S5-cells, followed by encapsulation and xenotransplantation (Figure 4A). Of note, a bulk global proteomics comparison of standard Matrigel-differentiated *HNF1*α^Δ/+^ (S7_HNF1α^Δ/+^) and wild-type cells (S7_WT) revealed a decrease of all islet hormone abundance in the *HNF1*α^Δ/+^ cells, especially somatostatin (Figure 4B). Moreover, the key β-cell marker PDX1 (Pancreas/duodenum homeobox protein 1), the neuroendocrine convertases PCSK1 and PCSK2 as well as the panendocrine marker CHGA exhibited less abundance in the S7_HNF1α^Δ/+^ cells. Interestingly, MAFB, a transcription factor with essential role for both human α- and β-cells displayed increased abundance in the HNF1A deficient samples. As expected, the upstream regulators analysis predicted the inactivation of HNF1A (Supplementary Figure S2K). Interestingly, the observed proteome landscape characterizing the *HNF1*α^Δ/+^ cells was also compatible with the inactivation of CDKN2A and HNF4A (Supplementary Figure S2K), two upstream regulators inferred above (Figure 3C) as activated in the healthy samples. Furthermore, the proteome signature of the *HNF1*α^Δ/+^ was consistent to the inactivation of PDX1 and PAX6 (Paired box protein Pax-6), critical transcription factors for islet development and cell fate selection (Supplementary Figure S2K).

Subsequently, the comparison of HNF1α^Δ/+_^cells and their WT counterparts after xeno-transplantation did not reveal a significant difference in the fraction of monohormonal glucagon^+^ cells (Figure 4D, first graph). In contrast, the proportion of the monohormonal insulin^+^ cells displayed a steep decline in the transplanted *HNF1*α^Δ/+^ cells (Figure 4D, middle graph). Importantly, the bihormonal fraction decrease observed in the transplanted control was not recapitulated in the HNF1α^Δ/+^-postTX cells (Figures 4C,D, last graph). Of note, the WT and HNF1A^Δ/+^ S7-populations exhibited a similar fraction of insulin^+^NKX6.1^+^ in two independent planar differentiation rounds (Supplementary Figure S2L).

These results suggest that *in vivo* exposure promotes the induction of a HNF1A-based mechanism, required for cell fate restriction toward single-hormonal identity possibly by regulating α-cell identity in β-cells.

We further investigated the role of HNF4A induction in mediating the observed *in vivo* effect on cell fate restriction by following the same experimental design as described above (Figures 4E,F). In this case we employed previously generated hiPSCs derived from patients (Vethe et al., 2017) bearing a dominant heterozygous p.Ile271fs mutation in the coding sequence of the *HNF4A* (Yamagata et al., 1996a; Fajans et al., 2001). Similar to the above rationale, we expect that the *in vivo* effect mediated induction of HNF4A-based mechanisms will be suboptimal following the transplant. Large-scale imaging microscopy revealed no significant change in the fractions of monohormonal glucagon^+^, or monohormonal insulin^+^ cells in *HNF4*α^Δ/+^ condition before xenotransplantation (Figure 4G). In contrast, following exposure to the *in vivo* environment, the percentage of monohormonal insulin^+^ cells was slightly increased in the transplanted *HNF4*α^Δ/+^ samples (Figure 4H). Moreover, the total fraction of bihormonal cells displayed a steep increase as compared to control transplanted cells (Figures 4F,I). These results indicate that HNF4A induction is required for the *in vivo* effect action on cell fate restriction.

Of interest, the fraction of hormone^+^ cells is significantly increased in the HNF4α^Δ/+^_postTX cells as compared to both the control and HNF1α^Δ/+^_postTX (Figure 4J). In contrast, in HNF1α^Δ/+^_postTX the proportion of hormone^+^ cells is lower than in both control and HNF4α^Δ/+^_postTX. This result advocates a possible role of the *in vivo* effect mediated HNF1A and HNF4A induction on endocrine program commitment.

Taken together these data suggest that the host’ *in vivo* effect requires the induction of both HNF1A and HNF4A in order to restrict and control cell fate selection.

## Discussion

Here we investigated the impact of short-term intraperitoneal xenotransplantation of encapsulated pancreatic progenitors. In this study we report that the brief exposure to the *in vivo* environment is insufficient for boosting the islet cell fraction, yet it is beneficial for cell fate selection as indicated by a steep decrease in the number of bihormonal cells and improved islet proteome signature. Of note, the proportion of bihormonal cells identified in the S7-population was more variable and slightly higher than the one usually reported in the literature (21.81 ± 13.2 vs. 20.2% previously reported in Rezania et al. (2014). This is probably a repercussion of using planar culturing conditions for the entire length of differentiation. We chose this approach in order to avoid the confounding effect caused by the switch to air-liquid interface (i.e. aggregation) characterizing the last two stages of the original differentiation protocol (Rezania et al., 2014).

Several works connected the bihormonal phenotype of the *in vitro* differentiated cells to their immaturity status. Consequently, the apparently subtle changes determining the cells to exclusively select and express a single islet hormone represent critical steps toward reaching the functionally mature status previously reported after long-term transplantation experiments (Rezania et al., 2014). It is tempting to speculate that the rapid initiation of the underlying cell fate restriction program following *in vivo* exposure could indicate an innate defense mechanism protecting against the inclusion of aberrant immature cells in adult host organisms. In addition, it should be stated that the significant islet-profile enhancement following transplantation does not necessarily involve a significant functional improvement. Due to the inherent difficulties in properly assessing the functionality of the encapsulated differentiating cells, this particular parameter requires further investigations. Nevertheless, it is, however, expected that a very brief *in vivo* exposure, such as the one used in this study, will be insufficient to promote any meaningful/detectable improvement in the functionality of the transplanted cells. Indeed, previous studies reported improved functionality and glucose stimulated insulin secretion in similar setups only after about 60 days post xenotransplantation (Rezania et al., 2014; Saber et al., 2018), thus involving longer exposure times.

Moreover, we believe this is the first attempt to comprehensively characterize the effect of the *in vivo* niche on the transplanted cells. On a cautionary note, it should be stated that the demultiplexing performed here is based on the premise that the three effects considered are independent variables. Essentially, it presumes that the encapsulation effect is similar *in vitro* and *in vivo* and thus, the differences between the two contexts are individually prompted by either the differentiation cocktail or the *in vivo* niche. Although this assumption is required for executing the analysis, the possibility that encapsulation effects vary between *in vitro* and *in vivo* environments should be kept in mind. Several previous studies reported increased starvation/hypoxia-based oxidative stress in encapsulated native human islets, as well as aggregated cells (Jacobs-Tulleneers-Thevissen et al., 2013; Weir, 2013; Barra and Tse, 2018; Evron et al., 2018), likely due to capsule’s size and density of the encapsulated structure. Yet, our analysis indicated that the observed changes in the redox balance are consistent with a more islet-like fingerprint, suggesting that single cells in suspension benefit of improved nutrient and oxygen exposure. In contrast, the proteins defining the neuro/neuritogenesis signature did not display a regulation pattern compatible with islet cell fate acquisition in response to the *in vivo* environment, suggesting the occurrence of additional processes following transplantation. Of interest, a substantial amount of research generated by the transplantation under the kidney capsules of native pancreatic islets/islet cells, reported the rapid vascularization (Brissova et al., 2004; Morini et al., 2007; Nyqvist et al., 2011) and innervation of the graft (Korsgren et al., 1992, 1993). One study showed that the sympathetic reinnervation paralleled the timeline of revascularization, with axons visible inside the islet grafts at 15 days post-transplant, closely associated with blood vessels (Rodriguez-Diaz et al., 2012). Although no study investigated the reinnervation of the encapsulated islets/cells, a similar mechanism might occur following xenotransplantation of encapsulated differentiating cells, possibly initiated by neuritogenic signals generated by the grafted cells.

Furthermore, the upstream regulators analysis predicted the activation of HNF1A transcription factor as top hit responsible for the proteome landscape observed in response *in vivo* environment. Interestingly, HNF1A is a transcriptional regulator with strong bounds with islet cell fate and functionality (Yamagata et al., 1996b; Wang et al., 1998, 2000; Haliyur et al., 2019). Of interest, a recent study on genetically modified ESCs via CRISPR/CAS9 system showed that loss of *HNF1A* leads to increased expression of α-cell markers (including glucagon), coupled with decreased expression of β-cells markers such as PAX4 (Paired box protein Pax-4). Furthermore, functional defects in both glycolysis and mitochondrial function were also detected upon *HNF1A* ablation (Cardenas-Diaz et al., 2019).

Here we show that the *in vivo* niche effect on transplanted cells requires both HNF1A and HNF4A induction. Our data suggest a potential cooperative, however, not overlapping role of the two effect regulators in restricting and maintaining the hormone expression choice. In this specific context, suboptimal HNF1A stimulation coupled with efficient *in vivo* HNF4A induction hinders the effect of *in vivo* niche on both cell identity restriction and, possibly, endocrine program commitment. Correspondingly, suboptimal HNF4A stimulation connected with the efficient *in vivo* HNF1A induction leads to an increased recruitment of endocrine cells characterized by an immature, bihormonal, profile. This interplay indicates that, besides the obvious importance of the optimal HNF1A and HNF4A induction, the ratio between the expression levels is also critical for mediating the *in vivo* effects on islet cell identity. Certainly, establishing HNF1A and HNF4A specific roles in controlling islet cell identity and especially endocrine commitment will require complex future *in vivo/in vitro* investigations into demultiplexing the intricate HNF1A-HNF4A axis. Moreover, the *in vivo* factor/s responsible for their induction are not known. Identification of these signal/s, besides improving the knowledge of the cellular and molecular basis of islet cell identity regulation, might benefit the current differentiation and regeneration strategies.

Based on the above results, we advance a model in which immediately after transplantation the *in vivo* environment promotes the islet profile in the differentiating pancreas progenitor cells, manifested by ameliorating their hormone expression phenotype. This probably occurs via an improved energy metabolism influencing the activity of specific epigenetic modifiers, such as MECP2. The underlying mechanism is at least partially dependent on the optimal levels of HNF1A and HNF4A, as decreased expression of these transcription factors not only cancel the observed confinement toward single hormone expression but also triggers an accumulation of immature bihormonal cells.

## Data Availability Statement

The datasets generated for this study can be found in the ProteomeXchange via the PRIDE (http://www.proteomexchange.org) partner repository with the dataset identifiers: PXD012704 and PXD015955.

## Ethics Statement

The studies involving human participants were reviewed and approved by the Norwegian Regional Committee of Medical and Health Research Ethics for hiPSCs (REK 2010/2295) and for human islets (REK 2011/426). All methods were carried out in accordance with the Helsinki Declaration. Informed consent was obtained from the healthy and MODY1/3 donors (skin fibroblasts) or from the relatives (organ donations). The patients/participants provided their written informed consent to participate in this study. The animal study was reviewed and approved by the Norwegian Animal Research Authority (FOTS IDs 8329 and 8423).

## Author Contributions

TL, ZS, HV, and LG performed the differentiation, encapsulation, and immunofluorescence staining. TL and HV prepared the samples for proteomic analyses. ZS, AM, and TL performed the mouse work, confocal imaging, and counting. YB generated the MODY3 lines by episomal reprogramming. SA and HS generated the human islet preparations. JP performed the TMT-labeling experiment and mass spectrometry analysis. TL and SC analyzed the proteomics data. HR provided the iPSC cell lines and access to the iPS facility. LG and SC conceived the experiments, interpreted the observations, and wrote the manuscript. All authors approved the final version of the manuscript.

## Conflict of Interest

The authors declare that the research was conducted in the absence of any commercial or financial relationships that could be construed as a potential conflict of interest.

## Footnotes

1 http://www.proteomexchange.org

2 www.qiagen.com/ingenuity

## References

[B1] Agulnick A. D. Ambruzs D. M. Moorman M. A. Bhoumik A. Cesario R. M. Payne J. K. (2015). Insulin-producing endocrine cells differentiated in vitro from human embryonic stem cells function in Macroencapsulation devices In Vivo. *Stem Cells Transl. Med.* 4 1214–1222. 10.5966/sctm.2015-0079 26304037 PMC4572906

[B2] Balboa D. Otonkoski T. (2015). Human pluripotent stem cell based islet models for diabetes research. *Best Pract. Res. Clin. Endocrinol. Metab.* 29 899–909. 10.1016/j.beem.2015.10.012 26696518

[B3] Barra J. M. Tse H. M. (2018). Redox-dependent inflammation in islet transplantation rejection. *Front. Endocrinol.* 9:175. 10.3389/fendo.2018.00175 29740396 PMC5924790

[B4] Bjorkhaug L. Sagen J. V. Thorsby P. Sovik O. Molven A. Njolstad P. R. (2003). Hepatocyte nuclear factor-1 alpha gene mutations and diabetes in Norway. *J. Clin. Endocrinol. Metab.* 88 920–931. 10.1210/jc.2002-020945 12574234

[B5] Bjørlykke Y. Søviknes A. M. Hoareau L. Vethe H. Mathisen A. F. Chera S. (2019). Reprogrammed cells display distinct proteomic signatures associated with colony morphology variability. *Stem Cells Int.* 2019:8036035. 10.1155/2019/8036035 31827534 PMC6885794

[B6] Bochenek M. A. Veiseh O. Vegas A. J. McGarrigle J. J. Qi M. Marchese E. (2018). Alginate encapsulation as long-term immune protection of allogeneic pancreatic islet cells transplanted into the omental bursa of macaques. *Nat. Biomed. Eng.* 2 810–821. 10.1038/s41551-018-0275-271 30873298 PMC6413527

[B7] Brissova M. Fowler M. Wiebe P. Shostak A. Shiota M. Radhika A. (2004). Intraislet endothelial cells contribute to revascularization of transplanted pancreatic islets. *Diabetes* 53 1318–1325. 10.2337/diabetes.53.5.1318 15111502

[B8] Bruin J. E. Erener S. Vela J. Hu X. Johnson J. D. Kurata H. T. (2014). Characterization of polyhormonal insulin-producing cells derived in vitro from human embryonic stem cells. *Stem Cell Res.* 12 194–208. 10.1016/j.scr.2013.10.003 24257076

[B9] Cardenas-Diaz F. L. Osorio-Quintero C. Diaz-Miranda M. A. Kishore S. Leavens K. Jobaliya C. (2019). Modeling Monogenic Diabetes using Human ESCs Reveals Developmental and Metabolic Deficiencies Caused by Mutations in HNF1A. *Cell Stem Cell* 25:273-289.e5. 10.1016/j.stem.2019.07.007 31374199 PMC6785828

[B10] Chera S. Herrera P. L. (2016). Regeneration of pancreatic insulin-producing cells by in situ adaptive cell conversion. *Curr. Opin. Genet. Dev.* 40 1–10. 10.1016/j.gde.2016.05.010 27266969 PMC5135655

[B11] Cigliola V. Ghila L. Thorel F. van Gurp L. Baronnier D. Oropeza D. (2018). Pancreatic islet-autonomous insulin and smoothened-mediated signalling modulate identity changes of glucagon(+) alpha-cells. *Nat. Cell Biol.* 20 1267–1277. 10.1038/s41556-018-0216-y 30361701 PMC6215453

[B12] Conrad E. Dai C. Spaeth J. Guo M. Cyphert H. A. Scoville D. (2016). The MAFB transcription factor impacts islet alpha-cell function in rodents and represents a unique signature of primate islet beta-cells. *Am. J. Physiol. Endocrinol. Metab.* 310 E91–E102. 10.1152/ajpendo.00285.2015 26554594 PMC4675799

[B13] Cyphert H. A. Walker E. M. Hang Y. Dhawan S. Haliyur R. Bonatakis L. (2019). Examining how the MAFB transcription factor affects islet beta-cell function postnatally. *Diabetes* 68 337–348. 10.2337/db18-0903 30425060 PMC6341297

[B14] Desgraz R. Herrera P. L. (2009). Pancreatic neurogenin 3-expressing cells are unipotent islet precursors. *Development* 136 3567–3574. 10.1242/dev.039214 19793886 PMC2761107

[B15] Dhawan S. Georgia S. Tschen S. I. Fan G. Bhushan A. (2011). Pancreatic beta cell identity is maintained by DNA methylation-mediated repression of Arx. *Dev. Cell* 20 419–429. 10.1016/j.devcel.2011.03.012 21497756 PMC3086024

[B16] Erickson B. K. Mintseris J. Schweppe D. K. Navarrete-Perea J. Erickson A. R. Nusinow D. P. (2019). Active instrument engagement combined with a real-time database search for improved performance of sample multiplexing workflows. *J. Proteome Res.* 18 1299–1306. 10.1021/acs.jproteome.8b00899 30658528 PMC7081948

[B17] Evron Y. Colton C. K. Ludwig B. Weir G. C. Zimermann B. Maimon S. (2018). Long-term viability and function of transplanted islets macroencapsulated at high density are achieved by enhanced oxygen supply. *Sci. Rep.* 8:6508. 10.1038/s41598-018-23862-w 29695723 PMC5917036

[B18] Fajans S. S. Bell G. I. Polonsky K. S. (2001). Molecular mechanisms and clinical pathophysiology of maturity-onset diabetes of the young. *N. Engl. J. Med.* 345 971–980. 10.1056/NEJMra002168 11575290

[B19] Friberg A. S. Stahle M. Brandhorst H. Korsgren O. Brandhorst D. (2008). Human islet separation utilizing a closed automated purification system. *Cell Trans.* 17 1305–1313. 10.3727/096368908787648100 19364068

[B20] Haliyur R. Tong X. Sanyoura M. Shrestha S. Lindner J. Saunders D. C. (2019). Human islets expressing HNF1A variant have defective beta cell transcriptional regulatory networks. *J. Clin. Invest.* 129 246–251. 10.1172/JCI121994 30507613 PMC6307934

[B21] Herrera P. L. (2000). Adult insulin- and glucagon-producing cells differentiate from two independent cell lineages. *Development* 127 2317–2322. 10804174 10.1242/dev.127.11.2317

[B22] Jacobs-Tulleneers-Thevissen D. Chintinne M. Ling Z. Gillard P. Schoonjans L. Delvaux G. (2013). Sustained function of alginate-encapsulated human islet cell implants in the peritoneal cavity of mice leading to a pilot study in a type 1 diabetic patient. *Diabetologia* 56 1605–1614. 10.1007/s00125-013-2906-2900 23620058

[B23] Korsgren O. Andersson A. Jansson L. Sundler F. (1992). Reinnervation of syngeneic mouse pancreatic islets transplanted into renal subcapsular space. *Diabetes* 41 130–135. 10.2337/diab.41.2.130 1346384

[B24] Korsgren O. Jansson L. Andersson A. Sundler F. (1993). Reinnervation of transplanted pancreatic islets. A comparison among islets implanted into the kidney, spleen, and liver. *Transplantation* 56 138–143. 10.1097/00007890-199307000-00026 7687393

[B25] Kramer A. Green J. Pollard J. Jr. Tugendreich S. (2014). Causal analysis approaches in Ingenuity pathway analysis. *Bioinformatics* 30 523–530. 10.1093/bioinformatics/btt703 24336805 PMC3928520

[B26] Kroon E. Martinson L. A. Kadoya K. Bang A. G. Kelly O. G. Eliazer S. (2008). Pancreatic endoderm derived from human embryonic stem cells generates glucose-responsive insulin-secreting cells in vivo. *Nat. Biotechnol.* 26 443–452. 10.1038/nbt1393 18288110

[B27] Kushner J. A. MacDonald P. E. Atkinson M. A. (2014). Stem cells to insulin secreting cells: two steps forward and now a time to pause? *Cell Stem Cell* 15 535–536. 10.1016/j.stem.2014.10.012 25517460

[B28] Legoy T. A. Ghila L. Vethe H. Abadpour S. Mathisen A. F. Paulo J. A. (2019). In vivo hyperglycemia exposure elicits distinct period-dependent effects on human pancreatic progenitor differentiation, conveyed by oxidative stress. *Acta Physiol.* 23:e13433. 10.1111/apha.13433 31872528 PMC7078042

[B29] Loo L. S. W. Lau H. H. Jasmen J. B. Lim C. S. Teo A. K. K. (2018). An arduous journey from human pluripotent stem cells to functional pancreatic beta cells. *Diabetes Obes. Metab.* 20 3–13. 10.1111/dom.12996 28474496

[B30] Melton D. A. (2016). Applied Developmental biology: making human pancreatic beta cells for diabetics. *Curr. Top. Dev. Biol.* 117 65–73. 10.1016/bs.ctdb.2015.11.013 26969972

[B31] Morini S. Brown M. L. Cicalese L. Elias G. Carotti S. Gaudio E. (2007). Revascularization and remodelling of pancreatic islets grafted under the kidney capsule. *J. Anat.* 210 565–577. 10.1111/j.1469-7580.2007.00717.x 17394557 PMC2375740

[B32] Nair G. G. Liu J. S. Russ H. A. Tran S. Saxton M. S. Chen R. (2019). Recapitulating endocrine cell clustering in culture promotes maturation of human stem-cell-derived beta cells. *Nat. Cell Biol.* 21 263–274. 10.1038/s41556-018-0271-274 30710150 PMC6746427

[B33] Nyqvist D. Speier S. Rodriguez-Diaz R. Molano R. D. Lipovsek S. Rupnik M. (2011). Donor islet endothelial cells in pancreatic islet revascularization. *Diabetes* 60 2571–2577. 10.2337/db10-1711 21873551 PMC3178280

[B34] Odorico J. Markmann J. Melton D. Greenstein J. Hwa A. Nostro C. (2018). Report of the key opinion leaders meeting on stem cell-derived beta cells. *Transplantation* 102 1223–1229. 10.1097/TP.0000000000002217 29781950 PMC6775764

[B35] Pagliuca F. W. Melton D. A. (2013). How to make a functional beta-cell. *Development* 140 2472–2483. 10.1242/dev.093187 23715541 PMC3666377

[B36] Pagliuca F. W. Millman J. R. Gürtler M. Segel M. Van Dervort A. Ryu J. H. (2014). Generation of functional human pancreatic β cells in vitro. *Cell* 159 428–439. 10.1016/j.cell.2014.09.040 25303535 PMC4617632

[B37] Petersen M. B. K. Azad A. Ingvorsen C. Hess K. Hansson M. Grapin-Botton A. (2017). Single-Cell gene expression analysis of a human ESC model of pancreatic endocrine development reveals different paths to Beta-cell differentiation. *Stem Cell Rep.* 9 1246–1261. 10.1016/j.stemcr.2017.08.009 28919263 PMC5639261

[B38] Petersen M. B. K. Goncalves C. A. C. Kim Y. H. Grapin-Botton A. (2018). Recapitulating and deciphering human pancreas development from human pluripotent stem cells in a dish. *Curr. Top. Dev. Biol.* 129 143–190. 10.1016/bs.ctdb.2018.02.009 29801529

[B39] Rezania A. Bruin J. E. Arora P. Rubin A. Batushansky I. Asadi A. (2014). Reversal of diabetes with insulin-producing cells derived in vitro from human pluripotent stem cells. *Nat. Biotechnol.* 32 1121–1133. 10.1038/nbt.3033 25211370

[B40] Rezania A. Bruin J. E. Riedel M. J. Mojibian M. Asadi A. Xu J. (2012). Maturation of human embryonic stem cell-derived pancreatic progenitors into functional islets capable of treating pre-existing diabetes in mice. *Diabetes* 61 2016–2029. 10.2337/db11-1711 22740171 PMC3402300

[B41] Rodriguez-Diaz R. Speier S. Molano R. D. Formoso A. Gans I. Abdulreda M. H. (2012). Noninvasive in vivo model demonstrating the effects of autonomic innervation on pancreatic islet function. *Proc. Natl. Acad. Sci. U.S.A.* 109 21456–21461. 10.1073/pnas.1211659110 23236142 PMC3535593

[B42] Saber N. Bruin J. E. O’Dwyer S. Schuster H. Rezania A. Kieffer T. J. (2018). Sex differences in maturation of human embryonic stem cell-derived beta cells in mice. *Endocrinology* 159 1827–1841. 10.1210/en.2018-2048 29420708

[B43] Tang S. C. Peng S. J. Chien H. J. (2014). Imaging of the islet neural network. *Diabetes Obes. Metab.* 16(Suppl. 1), 77–86. 10.1111/dom.12342 25200300

[B44] Thorel F. Nepote V. Avril I. Kohno K. Desgraz R. Chera S. (2010). Conversion of adult pancreatic alpha-cells to beta-cells after extreme beta-cell loss. *Nature* 464 1149–1154. 10.1038/nature08894 20364121 PMC2877635

[B45] Vegas A. J. Veiseh O. Gürtler M. Millman J. R. Pagliuca F. W. Bader A. R. (2016). Long-term glycemic control using polymer-encapsulated human stem cell-derived beta cells in immune-competent mice. *Nat. Med.* 22 306–311. 10.1038/nm.4030 26808346 PMC4825868

[B46] Velazco-Cruz L. Song J. Maxwell K. G. Goedegebuure M. M. Augsornworawat P. Hogrebe N. J. (2019). Acquisition of dynamic function in human stem cell-derived beta cells. *Stem Cell Rep.* 12 351–365. 10.1016/j.stemcr.2018.12.012 30661993 PMC6372986

[B47] Vethe H. Bjørlykke Y. Ghila L. M. Paulo J. A. Scholz H. Gygi S. P. (2017). Probing the missing mature β-cell proteomic landscape in differentiating patient iPSC-derived cells. *Sci. Rep.* 7:4780. 10.1038/s41598-017-04979-w 28684784 PMC5500592

[B48] Vethe H. Ghila L. Berle M. Hoareau L. Haaland O. A. Scholz H. (2019a). The effect of Wnt pathway modulators on human iPSC-derived pancreatic beta cell maturation. *Front. Endocrinol.* 10:293. 10.3389/fendo.2019.00293 31139151 PMC6518024

[B49] Vethe H. Legøy T. A. Abadpour S. Strand B. L. Scholz H. Paulo J. A. (2019b). Encapsulation boosts islet-cell signature in differentiating human induced pluripotent stem cells via integrin signalling. *bioRxiv [Preprint]* 31942009 10.1038/s41598-019-57305-x PMC6962451

[B50] Wang H. Antinozzi P. A. Hagenfeldt K. A. Maechler P. Wollheim C. B. (2000). Molecular targets of a human HNF1 alpha mutation responsible for pancreatic beta-cell dysfunction. *EMBO J.* 19 4257–4264. 10.1093/emboj/19.16.4257 10944108 PMC302029

[B51] Wang H. Maechler P. Hagenfeldt K. A. Wollheim C. B. (1998). Dominant-negative suppression of HNF-1alpha function results in defective insulin gene transcription and impaired metabolism-secretion coupling in a pancreatic beta-cell line. *EMBO J.* 17 6701–6713. 10.1093/emboj/17.22.6701 9822613 PMC1171015

[B52] Weir G. C. (2013). Islet encapsulation: advances and obstacles. *Diabetologia* 56 1458–1461. 10.1007/s00125-013-2921-2921 23636639

[B53] Yamagata K. Furuta H. Oda N. Kaisaki P. J. Menzel S. Cox N. J. (1996a). Mutations in the hepatocyte nuclear factor-4alpha gene in maturity-onset diabetes of the young (MODY1). *Nature* 384 458–460. 10.1038/384458a0 8945471

[B54] Yamagata K. Oda N. Kaisaki P. J. Menzel S. Furuta H. Vaxillaire M. (1996b). Mutations in the hepatocyte nuclear factor-1alpha gene in maturity-onset diabetes of the young (MODY3). *Nature* 384 455–458. 10.1038/384455a0 8945470

[B55] Yang C. Loehn M. Jurczyk A. Przewozniak N. Leehy L. Herrera P. L. (2015). Lixisenatide accelerates restoration of normoglycemia and improves human beta-cell function and survival in diabetic immunodeficient NOD-scid IL-2rg(null) RIP-DTR mice engrafted with human islets. *Diabetes Metab. Syndr. Obes.* 8 387–398. 10.2147/DMSO.S87253 26316789 PMC4548726

[B56] Zhou Q. Melton D. A. (2018). Pancreas regeneration. *Nature* 557 351–358. 10.1038/s41586-018-0088-80 29769672 PMC6168194

