# Supplementary material for: In vivo Environment Swiftly Restricts Human Pancreatic Progenitors Toward Mono-Hormonal Identity via a HNF1A/HNF4A Mechanism
Source: Front Cell Dev Biol. 2020 Feb 25;8:109. doi: 10.3389/fcell.2020.00109 (PMC7052484; doi:10.3389/fcell.2020.00109)
Supplement: Supplementary file 1 [file Data_Sheet_1.PDF]

Supplementary Material

in vivo environment swiftly restricts human pancreatic progenitors towards monohormonal identity via a HNF1A/HNF4A mechanism

Thomas Aga Legøy[1], Andreas F. Mathisen[1], Zaidon Salim[1], Heidrun Vethe[1], Shadab Abadpour[2,3], Joao A. Paulo[4], Hanne Scholz[2,3], Helge Ræder[1,5], Luiza Ghila[1] & Simona Chera[1,*]

[1]Department of Clinical Science, University of Bergen, Bergen, Norway

[2]Hybrid Technology Hub-Centre of Excellence, Faculty of Medicine, University of Oslo, Norway

[3]Institute for Surgical Research and Department of Transplant Medicine, Oslo University Hospital, Oslo, Norway

[4]Department of Cell Biology, Harvard Medical School, Boston, MA, USA

[5]Department of Pediatrics, Haukeland University Hospital, Bergen, Norway

*** Correspondence:**
Simona.Chera@uib.no
Tel: +47 55 97 12 62
Haukelandsbakken 15
5021 Bergen, Norway

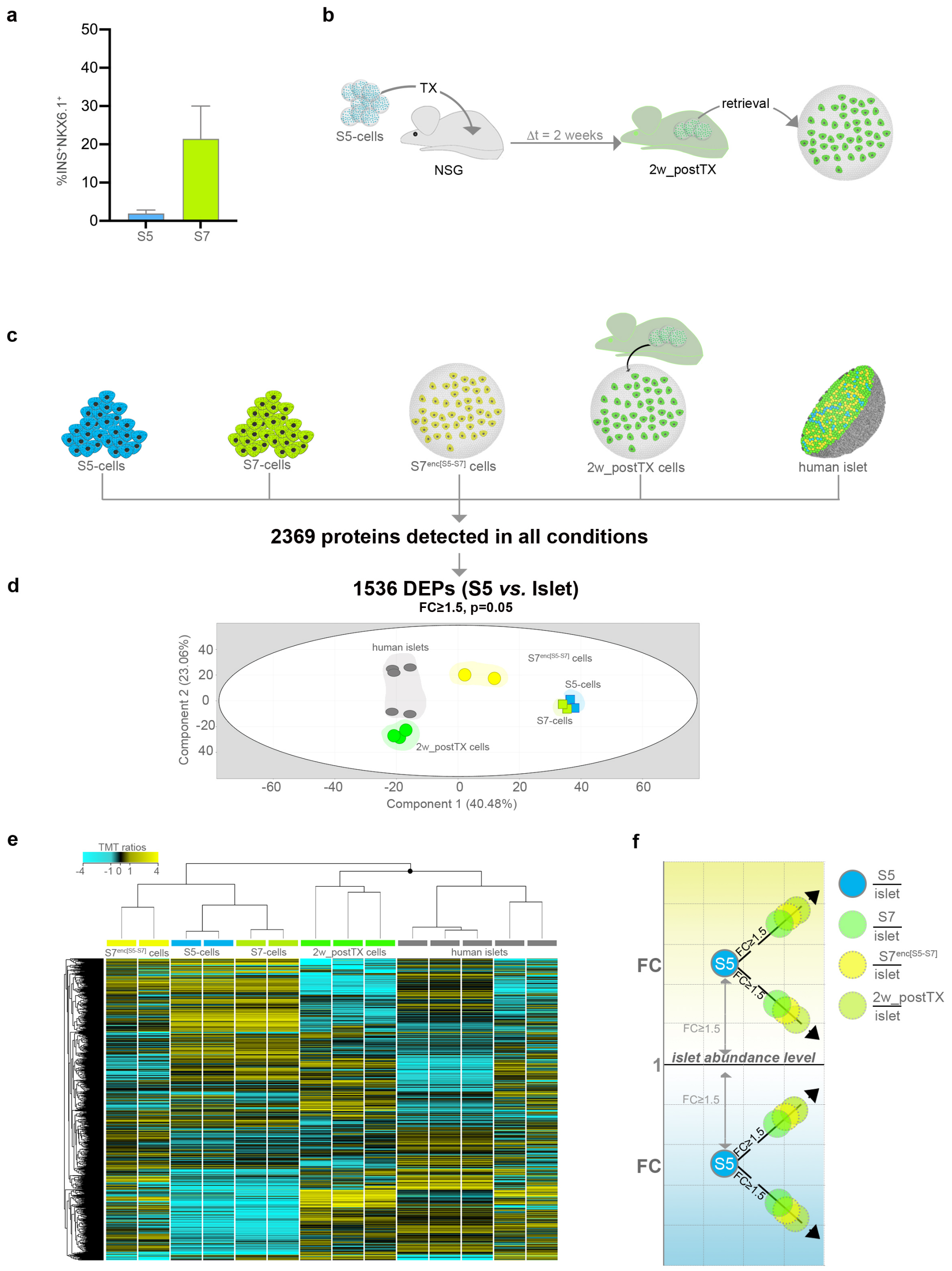

Supplementary Figure 1. a) Percentage of insulin and NKX6.1 co-expressing cells at stage 5 and stage 7 (mean±SEM) b) Experimental workflow. c) Analysis workflow of the conditions compared by TMT 11-plex proteomics over two plexes. d) PCA plot of the conditions analyzed. e) Hierarchical clustering of the normalized TMT-ratios (n=2,2,2,3,5). f) Scheme depicting the comparisons employed.

a

TOP 5 CANONICAL PATHWAYS		
Sirtuin Signaling Pathway	5.82E-16	inhibited (z:-1.567)
Mitochondrial Dysfunction	2.82E-12	
Oxidative Phosphorylation	3.58E-11	activated (z:2.132)
NRF2-mediated Oxidative Stress Resp.	5.11E-09	inhibited (z:-0.577)
Protein Ubiquitination Pathway	1.49E-08	

b

TOP PREDICTED ACTIVATED UPSTREAM REGULATORS

NAME	activation z-score
HNF1A	3.148
CST5	2.921
IL6	2.823
IL10RA	2.600
ERK	2.588
LEP	2.512

c

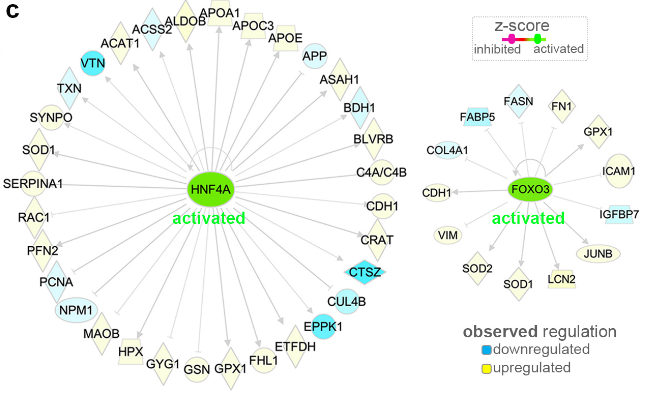

d

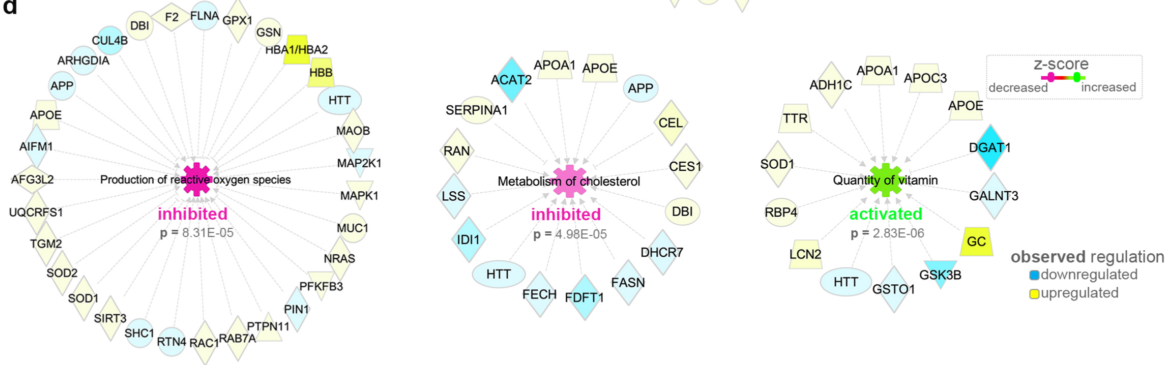

e

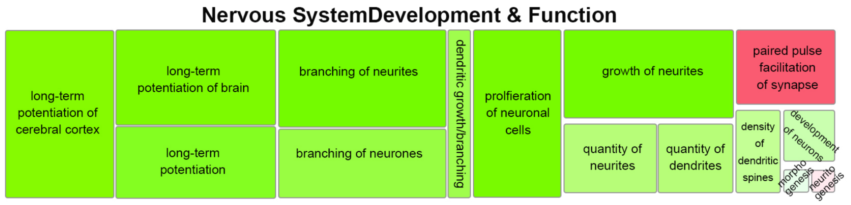

f

TOP 5 CANONICAL PATHWAYS	
Sirtuin Signaling Pathway	1.08E-11
Mitochondrial Dysfunction	3.05E-10
Oxidative Phosphorylation	5.46E-09
NRF2-mediated Oxidative Stress Resp.	4.75E-08
Protein Ubiquitination Pathway	2.87E-08

g

TOP PREDICTED ACTIVATED UPSTREAM REGULATORS

NAME	activation z-score
HNF1A	3.148
NFE2L2 (NRF2)	3.022
Esrra	2.985
CAB39L	2.828
LEP	2.721
SMARCA4	2.714

h

TOP PREDICTED INHIBITED UPSTREAM REGULATORS

NAME	activation z-score
RICTOR	-2.828
SOX2	-2.621
TSC2	-2.156
CIP2A	-2.000

i

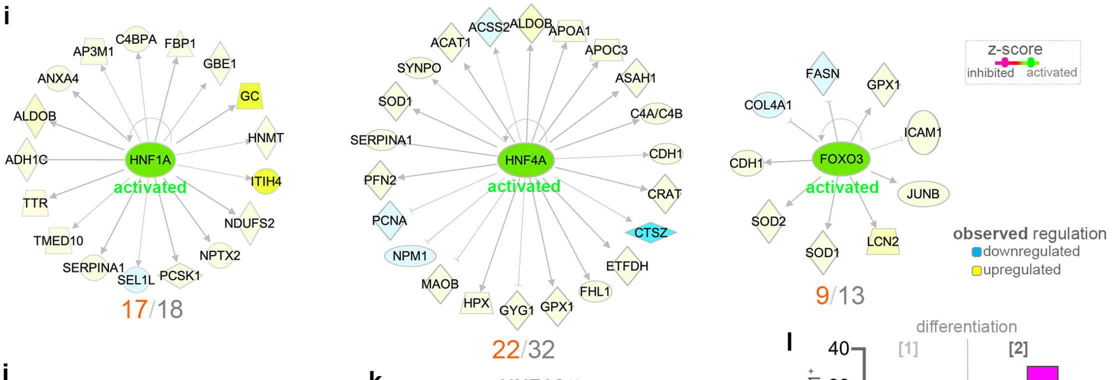

j

EPIGENETIC MODIFIERS (ISLET-PROMOTING EFFECT)

PROTEIN	FC	p-value
ASH2L	-10.07	0.0052
**MECP2**	1.74	0.0458
TRIM28	-2.34	0.0104
UBTF	-7.11	0.0119

k

HNF1A$^{\Delta/+}$

SELECTED RELEVANT PREDICTED INHIBITED UPSTREAM TRANSCRIPTION REGULATORS

NAME	activation z-score
PAX6	-3.818
HNF4A	-2.578
PDX1	-2.223
CDKN2A	-2.116
HNF1A	-2.104

l

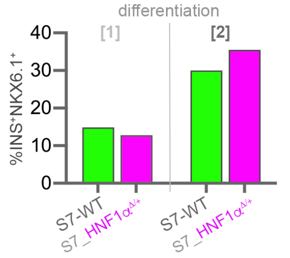

Supplementary Figure 2. a) IPA generated top canonical pathways characterizing the in vivo effect, b) Top predicted activated upstream regulators for the in vivo effect. c) HNF4A and FOXO3 target molecules observed regulated in the in vivo effect DEPs dataset. d) IPA-generated networks of the dataset DEPs underlying distinct top disease & function processes. e) the Nervous System Development & Function signature as inferred based on the proteins regulated by the in vivo effect. f) IPA generated top canonical pathways characterizing the in vivo effect protein subset displaying islet-promoting regulation. g, h) Top predicted (g) activated and (h) inactivated upstream regulators for the protein subset displaying islet-promoting regulation. i) HNF1A, HNF4A and FOXO3 target molecules observed regulated in the protein subset displaying islet-promoting regulation. j) Identified epigenetic regulators in the islet-promoting effect. k) Selected relevant predicted inactivated upstream transcription regulation in the S7_HNF1A$^{\Delta/+}$ cells. l) A side-by-side comparison of Ins^{+}Nkx6.1^{+} co-expressing fraction in WT control and HNF1A$^{\Delta/+}$ S7-cells in two distinct planar differentiation rounds.
